# Estimating the cold-induced brown adipose tissue glucose uptake rate measured by ^18^F-FDG PET using infrared thermography and water-fat separated MRI

**DOI:** 10.1038/s41598-019-48879-7

**Published:** 2019-08-26

**Authors:** Jonathan Andersson, Elin Lundström, Mathias Engström, Mark Lubberink, Håkan Ahlström, Joel Kullberg

**Affiliations:** 1 Section of Radiology, Department of Surgical Sciences, Uppsala University, Uppsala, Sweden; 2 Applied Science Laboratory Europe, GE Healthcare, Stockholm, Sweden; 3 Department of Medical Physics, Uppsala University Hospital, Uppsala, Sweden; 4 Antaros Medical, Mölndal, Sweden

**Keywords:** Medical research, Biomarkers

## Abstract

Brown adipose tissue (BAT) expends chemical energy to produce heat, which makes it a potential therapeutic target for combating metabolic dysfunction and overweight/obesity by increasing its metabolic activity. The most well-established method for measuring BAT metabolic activity is glucose uptake rate (GUR) measured using ^18^F-fluorodeoxyglucose (FDG) positron emission tomography (PET). However, this is expensive and exposes the subjects to potentially harmful radiation. Cheaper and safer methods are warranted for large-scale or longitudinal studies. Potential alternatives include infrared thermography (IRT) and magnetic resonance imaging (MRI). The aim of this study was to evaluate and further develop these techniques. Twelve healthy adult subjects were studied. The BAT GUR was measured using ^18^F-FDG PET during individualized cooling. The temperatures of the supraclavicular fossae and a control region were measured using IRT during a simple cooling protocol. The fat fraction and effective transverse relaxation rate of BAT were measured using MRI without any cooling intervention. Simple and multiple linear regressions were employed to evaluate how well the MRI and IRT measurements could estimate the GUR. Results showed that both IRT and MRI measurements correlated with the GUR. This suggest that these measurements may be suitable for estimating the cold-induced BAT GUR in future studies.

## Introduction

Human adipose tissue can be divided into white and brown adipose tissue (BAT), largely consisting of white and brown adipocytes, respectively. The main purpose of white adipose tissue is energy storage, while the main function of BAT is thermoregulation by non-shivering thermogenesis as a response to cold. Since the recent discovery of metabolically significant BAT in adult humans^1–4^ there has been significant research into BAT since its ability to expend energy could potentially be used to prevent or treat overweight, obesity, and metabolic dysfunctions such as type 2 diabetes^1–3,5^.

In adult humans, brown adipocytes are mixed with white adipocytes^2^ and are located in multiple depots^6^. For simplicity these depots may be called BAT depots, even though they contain a mixture of brown and white adipocytes. One of the largest^6^, and perhaps the most studied, depot is the supraclavicular depot. The supraclavicular depot is adjacent to two other BAT depots, the cervical and the axillary depots^6^.

When thermogenically active, BAT extracts fatty acids and glucose from the blood stream^4,7^. These molecules, as well as stored triglycerides and glucose, are combusted to provide heat^7^. One well-established method for estimating BAT metabolic activity is using ^18^F-fluorodeoxyglucose (^18^F-FDG) positron emission tomography (PET)/computed tomography (CT)^1–3^.

To measure the BAT metabolic activity the subjects are preferably exposed to cold since most persons do not have thermogenically active BAT under thermoneutral conditions^1,2^. This may be done using a fixed cooling protocol. However, this might give biased result, since e.g. a heavier person may be better isolated than a thinner person and therefore not as affected by the cold. Using an individualized cooling protocol, where the subjects are cooled as much as possible while avoiding shivering, is probably a better method for estimating the metabolic potential of the BAT.

By performing ^18^F-FDG PET scans it is possible to calculate e.g. thermogenically active BAT volume, total BAT radioactivity, mean or max BAT standardized uptake value, or BAT glucose uptake rate (GUR). Out of possible measurements, BAT GUR may be the superior measurement of BAT metabolic activity, since it is less sensitive to confounding factors such as body weight^8,9^.

^18^F-FDG PET/CT scans expose the subjects to potentially harmful levels of ionizing radiation and are expensive. The amount of ionizing radiation can be partially reduced by using a PET/magnetic resonance (MR) scanner instead of a PET/CT scanner. However, to enable large-scale or longitudinal prospective studies of BAT activity safe, reliable, and preferably cheap, methods are needed. Alternatives include measuring the skin temperature close to BAT, and measurements of BAT using MR scanners.

The temperature of the supraclavicular fossae (SCF) during cooling, or its temperature during cooling relative to other skin temperature measurements, has been found to be positively associated to measurements of cold-induced BAT metabolic activity performed using ^18^F-FDG PET^10–15^. The temperature may be measured either using attachable temperature probes^10,12,13^ or by infrared thermography (IRT)^11,14,15^. The correlations were likely due to the proximity between the SCF and the supraclavicular BAT depot. The metabolic activity of the BAT increases its temperature, which in turn increases the temperature of the nearby SCF, or at least prevents it from declining as much it otherwise would during cold exposure.

Both the fat fraction (FF)^16,17^ and the effective transverse relaxation rate (R_2_*)^16^ of BAT, measured using either MR spectroscopy^17^ or MR imaging (MRI)^16^, have been found to correlate negatively and positively, respectively, to measurements of cold-induced BAT metabolic activity performed using ^18^F-FDG PET^16,17^. However, other studies have failed to find such correlations^18,19^. The FF and R_2_* in BAT are lower and higher, respectively, than in white adipose tissue, likely because of differences in triglyceride content, mitochondrial abundance, and vascularization for white and brown adipocytes^20^. The metabolic capacity of a BAT depot is most likely linked to the concentration of brown adipocytes, which probably explains the correlations observed.

The purpose of the present study was to evaluate and further develop techniques using IRT and MRI for estimating cold-induced BAT metabolic activity by examining healthy adult subjects. The total GUR of the BAT was measured using ^18^F-FDG PET during individualized cooling and used as an estimate of BAT metabolic activity. The skin temperature of the SCF as well as a control region were measured using IRT, and the FF and the R_2_* of the BAT were measured using MRI. Simple and multiple linear regressions using the total GUR of the BAT as the dependent variable and IRT or MRI measurement(s) as independent variables were performed to evaluate how well these measurements could estimate the BAT metabolic activity.

## Subjects and Methods

### Study overview

**Figure 1:**
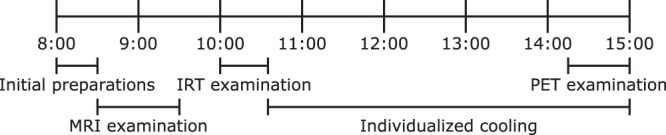
Schematic overview of the study protocol. Abbreviations used: MRI: magnetic resonance imaging, IRT: infrared thermography, PET: positron emission tomography.

Twelve healthy adult volunteers were included in the study, recruited mainly through advertisement. The study was approved by the Regional Ethical Review Board in Uppsala (diary no. 2015/108/2) and the Radiation Protection Committee in Uppsala (diary no. D16/10). All methods were carried out in accordance with the relevant guidelines and regulations. Only a subset of all data collected for the study was used in this manuscript. Only the pertinent parts of the study are described here. A schematic overview of the study is illustrated in Fig. 1.

Before examinations the subjects were asked to prepare according to the recommendations provided by the Brown Adipose Reporting Criteria in Imaging STudies (BARCIST 1.0)^8^. This included avoiding high-fat foods and caffeine for 24 h before the examination, fasting for at least 10 h before the examination, and avoiding strenuous activity for 48 h before the examination. Furthermore, the subjects were asked to avoid cold exposure immediately before the examinations, by e.g. dressing warmly while traveling to the site of examinations.

The following exclusion criteria were used: Having non-MR-compatible implant, or implants that were likely to negatively affect the quality of collected images. Having previously been exposed to large amounts of ionizing radiation. Being deemed to have their health risked if exposed to prolonged cooling. Using medication that could potentially interfere with the BAT function, such as β-adrenergic receptor agonists or beta blockers. Potential female subjects underwent a pregnancy test, and were excluded if found to be pregnant.

Subjects who had an ongoing infection, e.g. a cold, were not examined and asked to return at a later date. Subjects who had gained or lost more than 5% of their body mass during the last three months were asked to return at a later date when their weight had stabilized.

The subjects arrived at 8:00 for their examinations, and informed written consent was obtained. They answered some questions pertinent to the study and had some basic characteristics measured, including height and weight which were used to calculate body mass index (BMI). The subjects changed their clothing to a hospital gown, over which a vest with plastic tubing (CoolFlow Heavy Duty cooling vest, Polar Products, Stow, OH, USA) was placed. The vest was later on used to cool the subjects by means of cold water flowing through the tubing.

At 8:30 the subjects were placed in a clinical whole-body 3.0T PET/MR scanner (Signa PET/MR, General Electrics, Waukesha, WI, USA). Multiple MRI scans were performed, several of which were not used in this manuscript. One of the scans was used to calculate the BAT FF and R_2_* and one was used to calculate an alternative measure of the BAT FF and used as an anatomical reference for the PET scans. All scans were centred over the supraclavicular adipose depot. Scan protocols are described in detail below. The temperature in the scanning room was approximately 19 °C. No water was flowing through the tubing of the vest. The scanning was finished at 9:30.

After the scanning the vest was temporally removed. Measurements not used in this manuscript, were performed, which took a total of 30 minutes. At 10:00 the subjects sat down in a room with an air temperature of approximately 15 °C and were examined using IRT, details are described below, which took 35 minutes. After this an individualized cooling protocol took place. The vest was put back on with circulating water at approximately 4 °C. The water flowed from a chiller to the vest and back again through 7.5 m long thermally isolated plastic tubings. The subjects kept sitting in the room with the vest on until 13:15. If the subjects would start to shiver during this time period, the temperature of the circulating water would be increased until shivering stopped.

At 13:15 the subjects were again placed in the PET/MR scanner. The subjects still wore the vest and cold water kept flowing through it during the scanning. As before the temperature would be increased in case the subjects started to shiver. The temperature in the scanning room was approximately 18 °C. The first hour was spent doing the same examinations as during the first scanning session. After this an ^18^F-FDG PET scan was performed, this scan is described in detail below.

### Imaging protocols

#### PET scan

3 MBq/kg of FDG were injected simultaneously with the start of a 45 min dynamic scan with time frames 1 × 10, 8 × 5, 4 × 10, 2 × 15, 3 × 20, 2 × 30, 6 × 60, 4 × 150, and 5 × 300 s. The serum glucose level was measured before and after the dynamic FDG scan. All radioactivity injections were performed with a contrast medium injector as a fast bolus (10 ml at 1 ml/s followed by 30 ml saline at 2 ml/s). Images were created with a field-of-view (right-left × anterior-posterior × feet-head) = 500 × 500 × 247 mm^3^ and a reconstructed voxel size = 3.91 × 3.91 × 2.78 mm^3^ using a 3-dimensional reconstruction with time-of-flight ordered subset expectation maximization (3 iterations, 28 subsets) including point spread function recovery, applying all appropriate corrections for randoms, scatter etc. and a 5 mm Gaussian post-filter. Attenuation correction was based on a built-in water-fat MRI sequence performed during the PET scanning.

Image derived arterial whole blood time-activity curves were created by measuring the radioactive activity of the ascending aorta. The 5 inferiormost slices were of poor quality and not used for measuring the radioactive concentration, instead the concentration was measured over next the 5–10 inferiormost slices. In each of these slices a circlelike ROI centred over the centre of the ascending aorta with approximate diameter 1 cm was manually drawn in the frame in which the first pass of the radioactivity bolus was best visible. The ROIs together formed a VOI, and the mean radioactive activity of this VOI was used to create an arterial whole blood time-activity curves A plasma input curve was computed by multiplying the arterial whole blood time-activity curve by a typical mean plasma-to-arterial whole blood ratio of 1.1^21^.

FDG net uptake rate images were computed using a basis function implementation of the irreversible two-tissue compartment model, using one irreversible basis function and 50 basis functions with logarithmically spaced clearance rates between 0.02 and 1.0 min^−1^. These images were subsequently multiplied with the average of the serum glucose levels measured before and after the PET scan to obtain GUR maps, assuming a lumped constant, relating the FDG uptake to the glucose uptake, of 1. This assumption is based on the lumped constants of muscle^22^ and adipose tissue^23^ of humans, which have both been found to be close to 1.

#### Infrared thermography

As mentioned in Section 2.1 Study overview, the subjects were seated in a room cooled to approximately 15 °C. The room temperature and humidity were measured. An infrared camera (FLIR i60, FLIR Systems, Wilsonville, OR, USA) was used to measure the skin temperatures of the subjects. The camera was placed approximately 1.1 m from the subjects during imaging. Immediately before the imaging started the hospital gowns worn by the subjects were moved aside to expose the SCF so that they could be imaged. The images covered the faces of the subjects in addition to the SCF. One image was taken each minute during a total of 35 minutes. During the first 5 minutes no intervention was performed, for the next 5 minutes the subjects had their hands and feet submerged in 32 °C water, and for the last 25 minutes in 18 °C water.

Temperatures were calculated using The FLIR Atlas SDK for MATLAB. By providing the distance between the subject and the camera, the atmospheric and reflected (assumed to be the same as the atmospheric) temperatures (mean: 15 °C, standard deviation: 1 °C), the humidity (mean: 29%, standard deviation: 11%), and the emissivity of the skin, which was set to 0.98. In some cases the humidity measurement was not available, in these cases it was taken to be the average of all other cases.

#### MRI scans

Both MRI scans mentioned in Section 2.1 Study overview were 3D gradient echo sequences. The one used for calculating the BAT FF and R_2_* was a multi-echo scan, while the one used to calculate an alternative measure of the BAT FF and used an anatomical reference for the PET scans was a dual-echo scan. For all MRI scans a 19 channel receive only coil (Head Neck Unit, General Electrics, Waukesha, WI, USA) was used.

Multi-echo scan: The following scan parameters were used: right-left frequency encoding direction, TR/TE_1_/ΔTE = 13.8/1.70/0.65 ms, 15 unipolar echoes in 5 echo trains, flip angle = 4°, receive bandwidth =  ± 142.86 kHz, parallel imaging acceleration = 1.5 (ARC) in anterior-posterior and superior-inferior direction, field-of-view (right-left × anterior-posterior × feet-head) = 480 × 202 × 76 mm^3^, acquired/reconstructed voxel size = 1.00 × 1.00 × 2.00 mm^3^/0.94 × 0.94 × 2.00 mm^3^, no partial Fourier imaging was used, NEX = 1. The multi-echo scan used for the first subject of the study was slightly different. See the Supplementary Information for differences and how this was handled.

The scan took a total of 4 min and 52 s. The flip angle was chosen small to reduce T_1_-weighting. The subjects were instructed to breathe shallowly during the scan to reduce motion artefacts. No contrast agents were used.

In-house developed software was used to produce FF and R_2_* maps, as previously described^24^.

Dual-echo scan: The following scan parameters were used: right-left frequency encoding direction, TR/TE_1_/TE_2_ = 4.1/1.1/2.2 ms, 2 unipolar echoes in 1 echo train, flip angle = 12°, receive bandwidth =±166.67 kHz, parallel imaging acceleration = 2 (ARC) in anterior-posterior direction, field-of-view (right-left × anterior-posterior × feet-head) = 500 × 450 × 250 mm^3^, acquired/reconstructed voxel size = 1.95 × 2.36 × 5.00 mm^3^/1.95 × 1.95 × 2.50 mm^3^, partial Fourier imaging with 70% coverage was used, NEX = 1.

The scan took a total of 17 s. The subjects were instructed to hold their breath during the scan to reduce motion artefacts. No contrast agents were used.

Built-in software produced water and fat signal images. From these FF maps were calculated as fat signal/(water signal + fat signal).

### Image analysis

#### PET data

**Figure 2:**
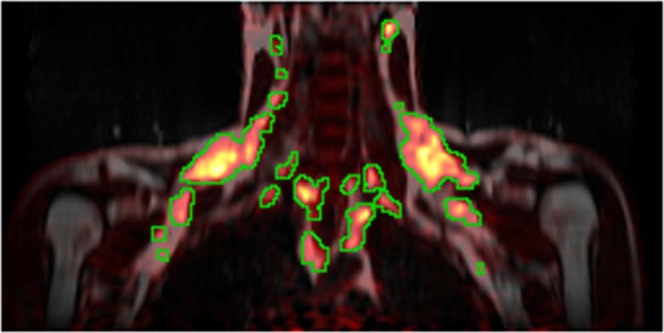
Coronal slice showing multiple BAT depots. MRI-derived fat signal image from the dual-echo scan in grayscale with the PET-derived GUR superimposed using the heat colourmap. The green lines delineate the areas over which the total BAT GUR was summed.

The GUR maps were interpolated into the resolution of the dual-echo images. The 6 inferior- and the 6 superiormost slices of the interpolated images were excluded from all measurements due to poor quality. The volume left for analysis spanned approximately from the upper border of heart up to the maxilla. All potential BAT within the field-of-view of the images, including the imaged parts of the cervical, supraclavicular, axillary, paraspinal, and mediastinal depots^6^, as well as the thoracic perivascular adipose tissues^25^, with a substantial GUR were segmented. This was done by creating an initial segmentation by including all areas with a GUR > 10 μmol/100 ml/min. This segmentation was imported and superimposed over the fat images from the dual-echo scan into Slicer 4.10.1^26^ and areas that were not belonging to a potential BAT depot, e.g. muscle, were manually removed. The total BAT GUR was calculated by summing the GUR over the entire segmentation. An example segmentation is shown in Fig. 2.

#### IRT data

**Figure 3:**
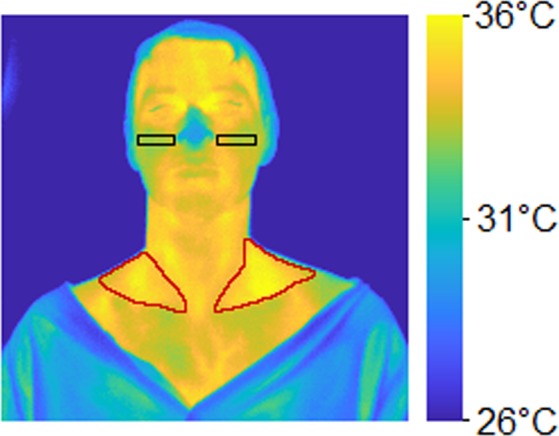
IRT image of a subject with hands and feet in cold water. Red lines delineate the SCF, black boxes delineate the PNR.

The mean temperatures over the first 5 and the last 10 minutes of the imaging of the SCF, henceforth referred to as SCF_neutral_ and SCF_cold_, respectively, were measured. Additionally, the mean temperatures over the same time periods of a region stretching horizontally from the wings of the nose out towards, but not including, the ears, which will be referred to as the paranasal region (PNR), where the activity of the BAT is not expected to directly affect the temperature, were measured. These temperature measurements will henceforth be referred to as PNR_neutral_ and PNR_cold_, respectively. Segmentations of the SCF and the PNR were manually performed. Example segmentations are shown in Fig. 3. The subject depicted in Fig. 3 gave informed written consent to have his image published in an online open-access publication.

#### MRI data

**Figure 4:**
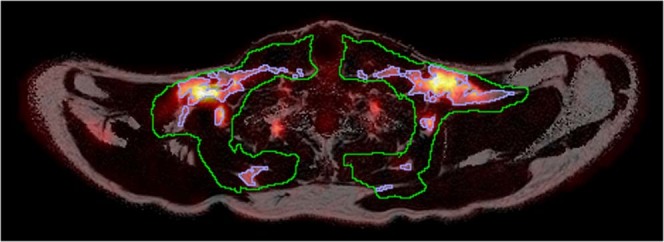
Axial slice showing the supraclavicular BAT depot. MRI-derived FF map from the multi-echo scan using the grayscale colourmap with the PET-derived GUR superimposed using the heat colourmap. The green delineation is the initial rough delineation and the blue delineation the area remaining after thresholding and erosion that was used to calculate BAT FF_15e_ and R_2_*. Note that data collected during the cooling is shown to illustrate the overlap between areas with a high GUR and the areas segmented to calculate BAT FF_15e_ and R_2_*.

Multi-echo scan: For the multi-echo scans the 3 inferior- and the 3 superiormost slices were excluded due to poor quality (caused by an imperfect slab profile). The volume left for analysis spanned approximately from the upper part of the thorax up to the larynx. A rough segmentation containing potential BAT, including the supraclavicular depot, parts of the cervical and axillary depots, as well as some other minor depots, was manually delineated as previously described^27^, after which the segmentation was refined by firstly removing voxels with a FF < 40%, secondly performing a 6-neighbourhood erosion, and finally removing voxels with a R_2_* > 120 s^−1^, also as in^27^ but with a different threshold for R_2_* due to differences between 1.5T and 3T MR systems. The mean FF and R_2_*, henceforth referred to as BAT FF_15e_ and BAT R_2_*, respectively, of the refined segmentations were measured. Only the multi-echo scans performed before cooling were used. An example segmentation is shown in Fig. 4.

Dual-echo scan: The refined segmentations from the multi-echo scans were transferred to the corresponding FF maps from the dual-echo scans performed before cooling. Fat masks were created by initially including all voxels with a FF ≥ 40% and thereafter performing a 6-neighbourhood erosion. The mean FF, henceforth referred to as BAT FF_2e_, of all voxels both in the transferred segmentations and the fat masks were calculated.

### Statistics

The IRT measurements (SCF_neutral_, SCF_cold_, PNR_neutral_, and PNR_cold_) and the MRI measurements (BAT FF_2e_, BAT FF_15e_, and BAT R_2_*) were used as independent variables with the total BAT GUR as the dependent variable in both simple and multiple linear regressions. Seven simple linear regressions were performed, one for each IRT and MRI measurement. Additionally, four multiple linear regressions were performed, using the SCF_neutral_ and PNR_neutral_, SCF_neutral_ and SCF_cold_, SCF_cold_ and PNR_cold_, or BAT FF_15e_ and BAT R_2_* as the independent variables. All continuous variables used in this manuscript were used in simple linear regressions against each other, with results presented in the Supplementary Information.

For all statistics, *p*-values < 0.05 were considered significant and *p*-values < 0.10 considered trends.

## Results

Basic characteristics, as well as IRT and MRI measurements and total BAT GUR of the subjects are shown in Table 1. Both males and females were included in the study. The subjects were mostly young (mean age 27.9 years). Most subjects were normal weight, but some were underweight or overweight/obese. All subjects except for one had a total BAT GUR > 0, although two additional subjects had an extremely low total BAT GUR (<0.4 µmol/min).

**Table 1:** Subject characteristics (n = 12).

Variable	Value
**Basic characteristics**	
Sex	7 males, 5 females
Age (years)	27.9 ± 6.2 (21.0–42.9)
Height (cm)	175.2 ± 8.1 (161.5–186.0)
Weight (kg)	73 ± 17 (55–108)
BMI (kg/m^2^)	23.7 ± 4.2 (18.3–34.3)
BMI classification	1 underweight, 8 normal weight, 2 overweight, 1 class I obesity
**PET**	
Total BAT GUR (µmol/min)	37 ± 42 (0–138)
**IRT**	
SCF_neutral_ (°C)	33.66 ± 0.75 (32.64–35.02)
SCF_cold_ (°C)	33.88 ± 0.65 (32.72–35.12)
PNR_neutral_ (°C)	32.16 ± 0.56 (31.28–32.88)
PNR_cold_ (°C)	31.43 ± 0.94 (30.15–33.02)
**MRI**	
BAT FF_2e_ (%)	83.3 ± 5.5 (73.3–91.1)
BAT FF_15e_ (%)	79.8 ± 7.1 (66.8–90.2)
BAT R_2_* (s^−1^)	44 ± 11 (31–62)

mean ± standard deviation (range).

**Figure 5:**
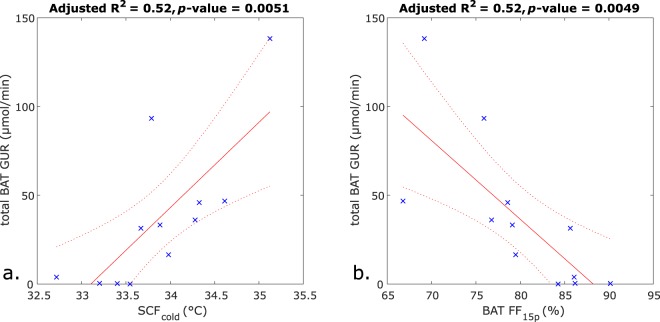
Simple linear regression plots with total BAT GUR as the dependent variable and (**a)** the SCF_cold_ temperature as the independent variable, or (**b**) the BAT FF_15e_ as the independent variable. Blue crosses represent observations, the solid red lines the fitted regressions, and the dotted red lines the 95% confidence bounds.

The results of the simple linear regressions are presented in Table 2 and the results of the multiple linear regressions are presented in Table 3. The simple linear regressions with SCF_cold_ and BAT FF_15e_ as independent variables are shown as graphs in Fig. 5a,b, respectively.

**Table 2:** Simple linear regressions with the total BAT GUR as the dependent variable.

Independent variable	Beta of independent variable	Intercept	Adjusted R^2^	*p*-value of independent variable
**IRT**				
SCF_neutral_ (°C)	31.35	−1018	0.25	0.055
SCF_cold_ (°C)	48.07	−1591	0.52	**0**.**0051**
PNR_neutral_ (°C)	−33.32	1109	0.12	0.15
PNR_cold_ (°C)	−23.82	786	0.22	0.072
**MRI**				
BAT FF_2e_ (%)	−5.53	498	0.48	**0**.**0075**
BAT FF_15e_ (%)	−4.44	391	0.52	**0**.**0049**
BAT R_2_* (s^−1^)	2.13	−57	0.22	0.072

Statistically significant *p*-values in **bold**.

**Table 3:** Multiple linear regressions with the total BAT GUR as the dependent variable.

Independent variable	Beta of independent variable	Intercept	Adjusted R^2^	*p*-value of independent variable
IRT, SCF_neutral_ & PNR_neutral_		122	0.50	
SCF_neutral_	34.98			**0**.**017**
PNR_neutral_	−39.25			**0**.**038**
IRT, SCF_neutral_ & SCF_cold_		−1632	0.53	
SCF_neutral_ (°C)	−28.15			0.30
SCF_cold_ (°C)	77.23			**0**.**029**
IRT, SCF_cold_ & PNR_cold_		−839	0.74	
SCF_cold_ (°C)	45.63			**0**.**0013**
PNR_cold_ (°C)	−21.32			**0**.**012**
MRI, BAT FF_15e_ & BAT R_2_*		831	0.59	
BAT FF_15e_ (%)	−8.33			**0**.**011**
BAT R_2_* (s^−1^)	−2.90			0.13

Statistically significant *p*-values in **bold**.

The results of all continuous variables in simple linear regressions against each other are presented in Supplementary Table S1.

**Figure 6:**
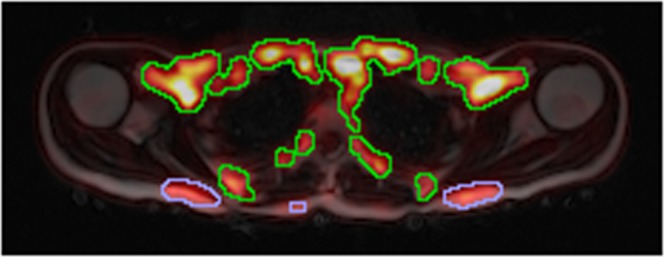
Axial slice showing the bilaterally symmetric FDG uptake in the posterior subcutaneous adipose tissue. MRI-derived fat signal image from the dual-echo scan in grayscale with the PET-derived GUR superimposed using the heat colourmap. The blue lines delineate the posterior subcutaneous adipose tissue areas with a GUR > 10 μmol/100 ml/min. The green lines delineate the remaining BAT areas with a GUR > 10 μmol/100 ml/min.

In the subject with the highest total BAT GUR a noteworthy amount of bilaterally symmetric GUR was noted in the posterior subcutaneous adipose tissue. This uptake was included in the total BAT GUR. The total GUR of the posterior subcutaneous adipose tissue was 5.0 µmol/min, equal to 3.6% of the total BAT GUR. An example image showing the uptake is show in Fig. 6.

## Discussion

We have shown that it is possible to estimate BAT metabolic activity during individualized cooling, measured as total GUR using ^18^F-FDG PET, both using IRT and MRI measurements. By examining the same subjects using both IRT and MRI protocols it was possible to directly compare these two methods regarding their ability to estimate BAT metabolic activity.

A significant positive correlation between SCF_cold_ and cold-induced BAT metabolic activity was found in the simple linear model. This is in line with previous studies in which measures of the SCF temperature after cooling have been found to correlate with cold-induced BAT activity measured using ^18^F-FDG PET^10–15^. However, in only two of these articles^10,15^ the SCF temperature during cooling alone, i.e. not relative to some other temperature measurement(s), was found to correlate with the BAT metabolic activity. In the other articles the SCF temperature during cooling relative to a control region^11^, to the pre-cooling SCF temperature^12,13^, or the change in SCF temperature during cooling relative the change in a reference region^14^ was instead used as the independent variable in the regressions. In three of these articles it is explicitly mentioned that there was no correlation found between the SCF temperature during cooling alone and BAT metabolic activity^11,12,14^.

Differences in results may be due to different measurements of BAT metabolic activity being used, for the significant correlations the volume was used in^10,11,13^, standardized uptake value in^10,12,15^, and GUR in^14^. Furthermore, different cooling protocols were used, performing individualized cooling using a vest or similar in^10,13^, individualized air-cooling in^12^, non-individualized air-cooling in^11^, and non-individualized cooling using a vest in^14,15^. Finally, somewhat different areas were covered by the PET scans in the different studies, and the temperature was not measured over the exact same region, which may have contributed to differences in the results.

In all of the studies mentioned above^10–15^ the same method for cooling was used for measuring the SCF temperature and activating the BAT for the ^18^F-FDG PET-scan. The current study differs compared to previous studies in that a cooling protocol in which the hands and feet of the subjects were first submerged in warm water, to enhance the feeling of cold when they thereafter were submerged in cold water, the temperature of which was not individualized, was used for the skin temperature measurements, while a separate individualized cooling protocol, using a cooling vest, was used to maximally activate the BAT without shivering during the PET-scan. The significant correlations that were obtained suggest that using a simple non-individualized cooling protocol may be enough to estimate individualized cooling-induced BAT metabolic activity, which could simplify future studies.

In the multiple linear model using both SCF_neutral_ and SCF_cold_ as independent variables, only SCF_cold_ contributed significantly to the model. The adjusted R^2^ of this model was similar to the model using only SCF_cold_ as an independent variable (0.53 vs. 0.52). This suggests that measuring the difference in SCF temperature after a period of cooling compared to before cooling is not essential.

The multiple linear regressions including both the SCF and PNR temperatures performed better than the corresponding models with only the SCF temperatures as an independent variables. This could suggest that the PNR temperature contains information useful for estimating BAT metabolic activity complimentary to that of SCF temperature. Previous studies have only used the difference between the SCF temperature and that of a control region in their regressions^11,14^. In this study multiple linear regressions were used, which allows for better fits.

In the two previous studies using a reference region either the lateral upper chest^11^ or the sternal apex^14^ was used. In this study we instead chose a region next to the nose as a reference, since chest hair on some of the men included in this study prevented accurate temperature measurements of the chest using IRT.

For both the simple and the multiple linear regressions using temperature measurements, the models using the temperature data collected during cooling performed better than the corresponding correlations using the data collected before cooling. This indicates that the cooling is important for better estimating the BAT potential metabolic activity. However, it is interesting to note that the model in which both SCF_neutral_ and PNR_neutral_ were used as independent variables, they both contributed significantly to the model.

Whenever a temperature measurement contributed significantly to a model, or would trend to do so, the SCF temperature would correlate positively with the cold-induced BAT metabolic activity, while the PNR temperature would correlate negatively. A possible reason for the correlations between the SCF temperature and BAT has already been mentioned in Section 1 Introduction. The negative correlations between PNR temperature and total cold-induced BAT metabolic activity might be explained by a reduced blood flow to the PNR during cooling, which serves to minimize the heat loss through the skin and is a defence mechanism against cold. Similarly, the increased metabolic activity of the BAT is a defence mechanism against cold. Since both responses are activated by the same stimulus, a correlation is plausible.

Significant negative correlations between BAT FF and BAT metabolic activity were found, in line with the results from three previous studies^15–17^, although no such correlation was found in two other studies^18,19^. However, no correlation between BAT R_2_* and BAT metabolic activity was observed, in contrast with the results of a previous study^16^ where a positive correlation was obtained, although the lack of a correlation was in line with the results of another study^19^. There was however a trend toward a positive correlation between BAT R_2_* and BAT metabolic activity in the simple linear regression in this study. In^16^ a separate scan was performed to measure R_2_*, supposedly optimized for this purpose, while in this study and in^19^ a single scan was used to measure both FF and R_2_*. The scan used in this study was not optimized for measuring R_2_*. It is possible that if a scan optimized for measuring R_2_* had been used, a statistically significant correlation between BAT R_2_* and BAT metabolic activity would have been observed^28^.

The adjusted R^2^ values of the simple linear regressions using BAT FF_2e_ and BAT FF_15e_ as independent variables were similar (0.48 and 0.52, respectively). This indicates that a long scan dedicated for accurate FF measurements is not necessary for estimating BAT metabolic activity, instead a shorter scan might suffice.

In the studies in which no correlation(s) were found between BAT FF^18,19^ and/or BAT R_2_*^19^ and BAT metabolic activity the BAT metabolic activity was estimated as standardized uptake value, volume, and total radioactivity^19^ or FDG activity concentration^18^. However, in this study and in^16,17^ dynamic ^18^F-FDG PET scans were used to calculate the BAT GUR which was used as a measure of BAT metabolic activity. The absence of correlations in^18,19^ in contrast to this study and in^16,17^ may in part be due to BAT GUR being a superior measurement of BAT for reasons mentioned in Section 1 Introduction. However, it should be noted that in^15^ a significant correlation was obtained, after adjusting for body weight and duration of cold exposure, despite estimating BAT metabolic activity using standardized uptake value. Most of these studies^16–19^ had in common that individualized cooling was performed using a vest or similar, as in this study. However, in^15^ non-individualized cooling using a vest during a non-standardized amount of time was performed.

Previously, the changes in BAT FF or R_2_* during cooling in relation to BAT metabolic activity have been studied^19^. This was not done in this study, partly to provide simple measurements for estimating cold-induced BAT activity for future studies. Furthermore, by taking the difference between different time points the measurements become more sensitive to noise and other imperfection. Additionally, the image quality was often slightly, in some cases substantially, worse during cooling for some subjects, likely due to shivering, even though measures were taken to prevent this.

In the multiple linear regression employing both BAT FF_15e_ and BAT R_2_* as independent variables BAT FF_15e_ remained statistically significant as an explanatory variable for BAT metabolic activity, while BAT R_2_* remained non-significant. The adjusted R^2^ of this model is slightly higher than the simple linear model employing only BAT FF_15e_ (0.59 vs. 0.52) indicating that BAT R_2_* might contain some information useful for estimating the BAT metabolic activity that is independent from the information provided by BAT FF. However, another study would be needed to ascertain this, perhaps preferentially utilizing a protocol optimized for measuring R_2_*^28^.

The best performing multiple linear regression using IRT measurements, i.e. the one using SCF_cold_ and PNR_cold_ as independent variables, resulted in a somewhat greater adjusted R^2^ compared to the multiple linear regression using MRI measurements, i.e. the one using BAT FF_15e_ and BAT R_2_* as independent variables (0.74 vs. 0.59). Regarding the simple linear regressions, the best performing model using an IRT measurement, i.e. the one using SCF_cold_ as the independent variable, resulted in a similar adjusted R^2^ compared to the best performing model using an MRI measurement, i.e. the one using BAT FF_15e_ as the independent variable (both 0.52). This suggests that using the IRT protocol presented in this study could may be as good as, or even superior, compared to the MRI protocol, when it comes to estimating the cold-induced BAT activity. IRT is also cheaper and easier to perform compared to an MRI examination, which is worth taking into consideration for future studies.

The coefficients of determination for the correlations with the BAT FF and IRT measurements as the independent variables found in this study were relatively high compared to previous studies on the same subject. This may in part be because several of the other studies did not use individualized cooling and/or used some other FDG-derived measure than GUR to estimate the BAT metabolic rate, which may lead to poorer correlations as discussed in Section 1 Introduction. Additionally, in this study most subjects were young, which could strengthen the relationships since it is possible that age otherwise could have played a more important role as a confounding factor.

It is interesting to note that one of the subjects had a noteworthy amount of bilaterally symmetric GUR in the posterior subcutaneous adipose tissue. The subject was relatively young, 26.6 years old, and it is possible that the uptake was due to a remnant of the interscapular BAT depot that is ubiquitous in infants. It has been shown using biopsies of deceased persons that individuals of this age may have some interscapular BAT left^29^.

It may be noted that the temperature of the SCF fossae probably mainly depend of the metabolic activity of the supraclavicular BAT, which may limit the predictive power of this measurement for estimating total BAT metabolic activity. Similarly, the multi-echo MRI measurements only covered parts of the BAT depots, mainly the cervical, supraclavicular, and axillary depots, while the dual-echo and the PET scan covered a larger volume, although still not all parts of all BAT depots. Despite this both IRT and both multi- and dual-echo MRI measurements did correlate with the cold-induced BAT metabolic activity. However, this may limit how well these measurements may predict the cold-induced BAT metabolic activity.

In this study the total GUR of BAT was used as a measurement of its metabolic activity, instead of GUR per mass unit that has been used in several previous studies^14,16,17^. This has the benefit of not having the total volume of depots that may contain BAT as a confounding factor. However, it should be noted that not all BAT was imaged in this study, which means that the total BAT GUR might be underestimated, and it may introduce a bias depending on how large part of the total BAT GUR that is imaged in each individual.

Finally, in this study the GUR was used as a measure of the BAT metabolic activity, which does not account for all metabolic activity. It has been estimated that in rats only approximately 12% of the total metabolism of BAT derives glucose extracted from the blood stream during submaximal BAT stimulation, and as little as 2% during maximal stimulation^30^. It is possible that there are individual differences in the percentage of the energy used by the BAT that derives from glucose extracted from the blood, which could introduce a bias.

## Conclusions

It is possible to estimate BAT metabolic activity during individualized cooling, measured as total BAT GUR from ^18^F-FDG-PET, both with BAT FF estimated from MRI during warm conditions and with temperature measurements performed using IRT during a non-individualized cooling protocol. The adjusted R^2^ values indicate that the IRT protocol used in this study is as good as, or possibly even superior to, the MRI protocol for the estimation of cold-induced BAT metabolic activity.

## Supplementary information

Supplementary information

## Data Availability

The datasets generated and analysed during the current study are available from the corresponding author on reasonable request.
