# Supplementary material for: Estimating the cold-induced brown adipose tissue glucose uptake rate measured by 18F-FDG PET using infrared thermography and water-fat separated MRI
Source: Sci Rep. 2019 Aug 26;9:12358. doi: 10.1038/s41598-019-48879-7 (PMC6710246; doi:10.1038/s41598-019-48879-7)
Supplement: Supplementary file 1 — Supplementary information [file 41598_2019_48879_MOESM1_ESM.pdf]

Estimating the cold-induced brown adipose tissue glucose uptake rate measured by ^{18}F-FDG PET using infrared thermography and water-fat separated MRI

Author list:

Jonathan Andersson[a], Elin Lundström[a], Mathias Engström[b], Mark Lubberink[a,c], Håkan Ahlström[a,d], Joel Kullberg[a,d]
[a] Section of Radiology, Department of Surgical Sciences, Uppsala University, Uppsala, Sweden
[b] Applied Science Laboratory Europe, GE Healthcare, Stockholm, Sweden
[c] Department of Medical Physics, Uppsala University Hospital, Uppsala, Sweden
[d] Antaros Medical, Mölndal, Sweden

Differences of the multi-echo scan of the first subject and how these were handled

The multi-echo scan of the first subject differed from the multi-echo scans of the later subjects in the following way: TR/TE_1/ΔTE = 22.5/2.06/1.07 ms, 15 unipolar echoes in 3 echo trains, flip angle = 7°, field of view (right-left × anterior-posterior × feet-head) = 480×202x56 mm^3. The subject was scanned again at a later visit using both the original protocol as well as the protocol used for the other subjects. Differences in BAT FF and R_2* for the two protocols were calculated, and these values were used the correct the values from the first visit.

Supplementary table

Supplementary Table S1. Simple linear regressions of all continuous variables against all other continuous variables.

Variable 1\Variable 2	Age (years)	Height (cm)	Weight (kg)	BMI (kg/m²)	Total BAT GUR (µmol/min)	$SCF_{neutral}$ (°C)	SCF_{cold} (°C)	$PNR_{neutral}$ (°C)	PNR_{cold} (°C)	BAT FF_{2e} (%)	BAT FF_{15e} (%)	BAT R_2* (s^{-1})
Age (years)	-	0.28/0.12	**0.0065/0.54**	**0.0040/0.58**	0.40/0.072	**0.015/0.46**	**0.021/0.43**	0.12/0.22	0.16/0.19	**0.028/0.40**	0.082/0.27	0.061/0.31
Height (cm)	-	-	**0.019/0.44**	0.28/0.12	0.51/0.044	0.58/0.032	0.70/0.015	0.82/0.0051	0.61/0.028	0.25/0.13	0.23/0.14	0.35/0.088
Weight (kg)	-	-	-	**0.000012/0.86**	0.057/0.32	**0.027/0.40**	**0.011/0.49**	0.93/0.00088	0.69/0.017	**0.0059/0.55**	**0.014/0.47**	**0.010/0.50**
BMI (kg/m²)	-	-	-	-	**0.039/0.36**	**0.0093/0.51**	**0.00086/0.69**	0.77/0.0088	0.84/0.0042	**0.0043/0.57**	**0.014/0.47**	**0.0054/0.56**
Total BAT GUR (µmol/min)	-	-	-	-	-	0.055/0.32	**0.0051/0.56**	0.15/0.20	0.072/0.29	**0.0075/0.53**	**0.0049/0.56**	0.072/0.29
$SCF_{neutral}$ (°C)	-	-	-	-	-	-	**0.000091/0.80**	0.70/0.016	0.97/0.00016	**0.0030/0.60**	**0.0011/0.67**	**0.00037/0.73**
SCF_{cold} (°C)	-	-	-	-	-	-	-	0.82/0.0052	0.81/0.0063	**0.00024/0.76**	**0.00035/0.74**	**0.00076/0.69**
$PNR_{neutral}$ (°C)	-	-	-	-	-	-	-	-	**0.00010/0.79**	0.65/0.022	0.44/0.061	0.70/0.015
PNR_{cold} (°C)	-	-	-	-	-	-	-	-	-	0.36/0.083	0.23/0.14	0.48/0.050
BAT FF_{2e} (%)	-	-	-	-	-	-	-	-	-	-	**0.00000048/0.93**	**0.00064/0.70**
BAT FF_{15e} (%)	-	-	-	-	-	-	-	-	-	-	-	**0.000066/0.81**
BAT R_2* (s^{-1})	-	-	-	-	-	-	-	-	-	-	-	-

p-value/R^2. Statistically significant correlations in **bold**.
